# Evaluating the Government Response to the House of Lords Inquiry Into Preterm Birth: A Commentary on the Gaps in Maternal Mental Healthcare

**DOI:** 10.1111/1471-0528.18250

**Published:** 2025-06-05

**Authors:** Semra Worrall, Laura Bridle, Karen Burgess, Naomi H. Carlisle, Jenny Carter, Soo Downe, Laura A. Magee, Daghni Ragasingam, Andrew H. Shennan, Rachel M. Tribe, Peter von Dadelszen, Victoria Fallon, Paul Christiansen, Asma Khalil, Sergio A. Silverio

**Affiliations:** ^1^ Department of Psychology Institute of Population Health, University of Liverpool Liverpool UK; ^2^ HELIX Service, Maternal Mental Health Services King's College Hospital NHS Foundation Trust London UK; ^3^ PETALS: The Baby Loss Counselling Charity Cambridge UK; ^4^ Division of Methodologies Florence Nightingale Faculty of Nursing, Midwifery & Palliative Care, King's College London London UK; ^5^ Department of Women & Children's Health, School of Life Course & Population Sciences King's College London London UK; ^6^ School of Nursing and Midwifery University of Central Lancashire Preston UK; ^7^ Maternity Services St. Thomas' Hospital, Guy's and St. Thomas' NHS Foundation Trust London UK; ^8^ Molecular & Clinical Sciences Research Institute City St. George's, University of London London UK; ^9^ Department of Women's and Children's Health, Institute of Life Course and Medical Sciences University of Liverpool Liverpool UK

**Keywords:** clinical research, health services research, preterm labour

## 1 Overview

Preterm birth is a significant public health concern. In England, 8.1% of all births in 2023 were classified as premature [1]. The psychological impact of preterm birth on parents has become of increasing interest to researchers. However, the long‐term psychological impact on preterm babies, the effect prematurity has on wider family units, and the effect of working with preterm babies on healthcare professionals remains under investigated. Preterm birth remains the leading cause of neonatal death in the UK [2], but with improvements in the survival rates of extremely preterm infants, and their associated short‐ and long‐term developmental challenges, mothers and the wider family can experience profound psychological difficulties, particularly postpartum anxiety [3].

In 2017, the UK Government aimed to reduce both the rate of preterm birth to 6% by 2025 — and its associated adverse consequences. In 2024, the House of Lords commissioned an inquiry into the incidence and impact of preterm birth, to identify priority areas for research and clinical intervention. This report, published in November 2024 [4], confirmed that the government's targets are not on track, and made numerous recommendations. In January 2025, the UK Government responded [5]. This commentary critically assesses the governmental response to Recommendation 7, regarding equitable access to neonatal outreach and perinatal mental health services for all families who experience preterm birth.

## 2 Neonatal Outreach Services

Neonatal outreach services comprise multidisciplinary teams to support families following their infants' discharge home from a neonatal unit. These services are essential to improve outcomes such as minimising separation between baby and parents, reducing the financial burden, and a reduction of NICU stay [6]. A systematic review from 2023 has highlighted the benefits of such services, including reducing hospital re‐admission rates and improving breastfeeding rates and infant weight gain when compared with standard care [7]. However, we should treat these findings with some degree of caution, given the review highlights the current evidence‐base is rooted in research of variable quality. Further, given there is no national standard of practice for neonatal outreach services [7], more research is required to evaluate its effectiveness, particularly for long‐term maternal mental health outcomes.

The British Association of Perinatal Medicine [BAPM] recently published draft guidance for Neonatal Outreach Services [7], including a recommendation that all babies who have received specialist neonatal care have equitable, seven‐day‐per‐week access to Neonatal Outreach Services, to support the transition from hospital‐to‐home, which can be a period of heightened vulnerability for mental health difficulties [8]. However, this has yet to be implemented and would require significant education, training, and funding to develop an effective support system at national and regional levels. The guidance further recommends additional research to identify the most appropriate screening tools for use during the perinatal period; much of our work to date has focused on the development of a preterm‐ and NICU‐specific tool for measuring postpartum anxiety [9], which is currently in the final stages of development.

The response from the government to the House of Lord's Inquiry highlights the lacuna which remains with respect to the importance of mental health amongst mothers of preterm babies. The persistent undervaluing of its importance by the Government trickles down into policymakers, and eventually diminishes the importance of mental health related to preterm birth on the frontline of NHS services in this temporally unique transition. Further, there is an underestimation of the time and resources required to standardise essential programmes, such as Neonatal Outreach Services, across England and Wales. This is especially pertinent considering ethnic minority women may experience worse outcomes following a preterm birth, and may face difficulty accessing healthcare [10]. Recent evidence has suggested in order to improve maternal mental health after preterm birth amongst ethnic minority communities, healthcare providers must leverage specialist mental health services, and engage in cultural sensitivity [11]. This suggests healthcare providers must acknowledge the fact that Black, Asian, and Minority Ethnic women are still more likely to die due to complications in pregnancy and childbirth in Western hospital settings than their White counterparts [12]. Furthermore, it has been recommended that staff in maternity and children's health services should engage in cultural humility extending beyond race to culture and class in order to reduce frontline racial and ethnic tensions and improve care provision [13]. At a fundamental level, all women who access maternity care should feel able to communicate their needs and anxieties around their pregnancy and birth [14]. A successful way which has already been proven to improve communication amongst non‐native speakers is to leverage multi‐lingual staff and effective, professional translation services [15]. This is something which has been consistently highlighted as requiring specific attention [16], and should remain at the forefront of government, research and policy efforts.

## 3 Perinatal Mental Health Services

In their response to the House of Lords report, the Government argues that substantial progress has been made in providing perinatal mental health services, which are available to mothers who give birth prematurely. Specifically, they highlight the commissioning of 159 beds in mother and baby units [MBUs] across England, the provision of 41 maternal mental health services, and a 98% increase in those able to access a specialist community perinatal mental health service or a maternal mental health service. Yet, it is well established that there are large disparities in provision and access to care [17], and one service/unit has already closed due to funding issues [18]. The initial evaluation of early implementers and fast followers [17] highlights variations in service provision and the risk of unmet needs if existing gaps are not addressed elsewhere in the care pathway. As services continue to evolve, the latest progress report from the Maternal Mental Health Alliance [18] underscores the scale of this challenge; and that some provision would be better than no provision as was previously the case. It concludes whilst most areas in England have some kind of perinatal mental health service, gaps and overwhelming demand of care mean that there are not enough resources to meet the current demand. Similarly, whilst specialist antenatal care can be provided to women who have either experienced a previous preterm birth or have relevant risk factors which increase the likelihood of preterm birth, mental health is not routinely screened in these clinics [19]. Specific services are offered to women who have experienced perinatal bereavement, birth trauma, and tokophobia, as well as those who have experienced care proceedings, but very few services offer the specialised care women who have experienced preterm birth or neonatal intensive care unit admission require. Whilst the Government may argue that the increasing provision of neonatal practitioner psychologists may address this gap, they specialise in support whilst on the neonatal unit. Yet, many women and parents may need specialist mental health services once they are home with their babies, and for many months and years afterwards. Detail is lacking on how equitable access to these services will be ensured and how this will be funded. It will be difficult to act to improve outcomes if this is not further researched.

## 4 Recommendations Following the Government's Response to the House of Lord's Inquiry Into Preterm Birth

Policy gaps can be addressed by implementing the evidence‐based research findings relating to preterm birth and maternal mental health which already exist [11]. Practical issues such as funding and implementation of new ways of working will need to be met with investment from the Government to ensure the highest possible quality care is not only sustained, but ever improving. With respect to accountability, whilst local, regional, and national entities must work together and to a common goal, it has been suggested that maternity care services of the future in the UK will benefit from local co‐production of services to meet the needs of the local, diverse populations of this country [20]. Mental health following preterm birth and/or NICU admission has been under‐prioritised and under‐researched [21]. One of the new strategic priority areas of the Tommy's National Centre for Preterm Birth Research is psychological support. Below, we provide recommendations for research, policy and clinical practice change to highlight the need for increased mental health care provision for families after experiencing preterm birth or NICU admission:

1. Health visitors, midwives, and doctors must receive specific training in the mental health and psychosocial consequences of preterm birth and NICU admission.
2. Increased funding is needed for mental health interventions during and after NICU admission, including specific details from the Government as to how neonatal practitioner psychologists can be accessed equitably both for those in hospital and those who have been able to take their babies home.
3. Specialist perinatal mental health services are needed to support families whose babies have spent time in NICUs and to recognise them as a vulnerable group who may require increased, specialised mental healthcare in the medium‐ and longer‐term.
4. Mental health support should be offered as part of routine postnatal care for these women, and that care should continue after their babies are transferred home.
5. There needs to be a specific postnatal care pathway for women, babies, and families who spend extended periods of time in the NICU so that their scheduled appointments are still met and attended.
6. Clear information is needed for families about where to seek help post‐discharge.
7. There should be a focus on the development of perinatal‐specific measures of mood, both for research and clinical purposes.

Actions which can be taken in‐line with current policy priorities in the shortterm can be to leverage the specialist services for both perinatal mental health and preterm birth which are already functioning as part of the NHS provision. Furthermore, postnatal care must be enhanced and delivered equally across all mothers, paying particular attention to those of preterm babies. Longer‐term ambitions of the healthcare service should be focused on the implementation of validated tools, which are already developed or in progress, to offer the most effective identification, screening, and management of perinatal mental health conditions from conception up to and including the year postpartum. All of this screening and care must take into account the unique challenges faced by mothers of preterm infants, which the Government's response to the House of Lords inquiry currently appears to neglect.

## Author Contributions

Conceptualization: S.W., S.A.S. Writing – original draft: S.W. Writing – review and editing: S.A.S., L.B., K.B., N.H.C., J.C., S.D., L.A.M., D.R., A.H.S., R.M.T., P.v.D., V.F., P.C., A.K. Supervision: S.A.S., A.K., V.F., P.C. Project administration: S.W.

## Ethics Statement

The authors have nothing to report.

## Conflicts of Interest

The authors declare no conflicts of interest.

## Data Availability

Data sharing is not applicable to this article as no new data were created or analyzed in this study.
